# CLAE方案治疗难治/复发急性T淋巴细胞白血病/淋巴瘤的疗效和安全性：一项前瞻性、多中心、单臂临床研究

**DOI:** 10.3760/cma.j.cn121090-20250123-00043

**Published:** 2025-11

**Authors:** 艳 李, 弦 张, 秀华 孙, 嘉 宋, 嵘 张, 萍 杨, 伟 万, 菲 董, 继军 王, 红梅 景

**Affiliations:** 1 北京大学第三医院，北京 100191 Peking University Third Hospital, Beijing 100191, China; 2 河北燕达陆道培医院，廊坊 065206 Hebei Yanda Lu Daopei Hospital, Langfang 065206, China; 3 大连医科大学附属第二医院，大连 116023 The Second Hospital of Dalian Medical University, Dalian 116023, China; 4 天津医科大学总医院，天津 300052 Tianjin Medical University General Hospital, Tianjin 300052, China; 5 中国医科大学附属盛京医院，沈阳 110004 Shengjing Hospital of China Medical University, Shenyang 110004, China

**Keywords:** 前体T细胞淋巴母细胞白血病淋巴瘤, 复发, 难治, 克拉屈滨, 阿糖胞苷, 依托泊苷, Precursor T-cell lymphoblastic leukemia-lymphoma, Recurrence, Refractory Diseases, Cladribine, Cytarabine, Etoposide

## Abstract

**目的:**

探讨CLAE（克拉屈滨+阿糖胞苷+依托泊苷）方案治疗难治/复发急性T淋巴细胞白血病/淋巴瘤（R/R T-ALL/LBL）的疗效和安全性。

**方法:**

通过前瞻性、多中心、单臂临床研究，采用CLAE方案治疗R/R T-ALL/LBL患者或临床研究外行同情用药。收集2019年3月至2024年8月期间5个中心入组临床研究的18例患者以及北京大学第三医院7例同情用药患者临床资料并进行随访，评估CLAE方案再诱导治疗1个周期及2个周期的客观缓解率（ORR）、完全缓解（CR）率、部分缓解（PR）率；分析桥接allo-HSCT治疗情况及无进展生存（PFS）、总生存（OS）、治疗期间不良反应。

**结果:**

25例患者确诊T-ALL/LBL的中位年龄为29（13～63）岁，男性17例。可评价的24例患者CR率为33.3％，17例入组临床研究的患者CR率达41.2％。24例患者中位OS期和PFS期分别为199（46～1 310）d和49（28～1 310）d，6个月、1年、2年累积OS率分别为（52.1±10.2）％、（29.7±9.3）％、（27.1±9.1）％，累积PFS率分别为（32.6±9.6）％、（24.9±8.9）％、（23.8±8.7）％。CLAE缓解组（CR+PR，8例）中位OS期和PFS期均未达到，6个月、1年、2年累积OS率分别为（86.8±12.0）％、（78.3±14.6）％、（72.9±15.7）％，累积PFS率分别为（86.4±12.1）％、（74.8±15.3）％、（72.9±15.7）％。治疗相关不良反应主要是血液学不良反应，无治疗相关死亡。7例CR患者桥接allo-HSCT，其中5例持续缓解存活。

**结论:**

CLAE方案治疗R/R T-ALL/LBL安全有效，可使患者获得CR后行allo-HSCT治疗，改善患者预后。

## Abstract (en)

**Objective:**

To evaluate the efficacy and safety of the CLAE（cladribine + cytarabine + etoposide）regimen in refractory/relapsed T cell acute lymphoblastic leukemia/lymphoma（R/R T-ALL/LBL）.

**Methods:**

Patients with R/R T-ALL/LBL received the CLAE regimen in a prospective, multicenter, single-arm clinical study or compassionate use. From March 2019 to August 2024, data from 25 patients（18 in the study across five centers and 7 receiving compassionate treatment in Peking University Third Hospital）were collected. Outcomes included objective response rate, complete response（CR）rate, partial response（PR）rate after 1–2 cycles, bridging to allo-HSCT, progression-free survival（PFS）, overall survival（OS）, and treatment-related adverse effects.

**Results:**

Median age was 29 years（range, 13–63）; 17 were male. Among the 24 evaluable patients, CR rate was 33.3％ overall and 41.2％ among enrolled patients. Median OS and PFS time were 199（46–1 310）and 49（28–1 310）days, respectively. Cumulative OS rate at 6 months, 1 year, and 2 years was（52.1±10.2）％,（29.7±9.3）％, and（27.1±9.1）％, respectively; cumulative PFS rate was（32.6±9.6）％,（24.9±8.9）％, and（23.8±8.7）％, respectively. Among patients achieving CR or PR（8 cases）, median OS and PFS were not reached. Cumulative OS rate at 6 months, 1 year, and 2 years was（86.8±12.0）％,（78.3±14.6）％, and（72.9±15.7）％, respectively, and the cumulative PFS rate was（86.4±12.1）％,（74.8±15.3）％, and（72.9±15.7）％, respectively. Adverse events were mainly hematologic; no treatment-related mortality occurred. Seven patients achieving CR were bridged to allo-HSCT, with 5 remaining in continuous remission.

**Conclusion:**

The CLAE regimen is safe and effective for R/R T-ALL/LBL, facilitating CR as a bridge to allo-HSCT and potentially improving patient prognosis.

急性T淋巴细胞白血病/淋巴瘤（T-ALL/LBL）是来源于前体T细胞的血液系统恶性肿瘤，约占成人ALL/LBL的20％[1]–[3]。尽管多药联合化疗方案的早期缓解率为90％左右，但仍有高达40％的患者在2年内复发，难治/复发（R/R）患者远期生存不足10％[1],[4]。allo-HSCT已被证实可改善R/R T-ALL/LBL患者的生存[1],[5]–[6]，但需要患者移植前再诱导治疗达到完全缓解（CR）。然而，R/R T-ALL/LBL的最佳再诱导治疗方案尚无定论[1]–[2]。应用于R/R T-ALL/LBL治疗的新药有限，多数处于临床研究阶段，可及性差，且疗效有待进一步验证[4],[7]。因此，临床亟需探讨新的治疗方案，使R/R T-ALL/LBL患者获得更高的缓解率进而行allo-HSCT，改善预后。

克拉屈滨作为一种脱氧腺苷类似物，可选择性地引起淋巴细胞凋亡。该药能透过血脑屏障，且与阿糖胞苷有协同作用，同时具有去甲基化作用，可通过表观遗传学调控等提高抗肿瘤疗效[8]–[9]。克拉屈滨已广泛用于难治复发白血病、淋巴瘤的治疗，具有良好的疗效和安全性[10]–[12]。本研究采用CLAE（克拉屈滨+阿糖胞苷+依托泊苷）方案治疗25例R/R T-ALL/LBL患者，旨在系统评估该方案的临床疗效和安全性。

## 病例与方法

1. 病例：纳入自2021年4月至2024年8月于一项前瞻性、多中心、单臂临床试验注册研究（NCT04679506）中接受CLAE方案治疗的R/R T-ALL/LBL患者18例，其中北京大学第三医院9例、河北燕达陆道培医院6例、大连医科大学附属第二医院1例、天津医科大学总医院1例、中国医科大学附属盛京医院1例。主要入选标准：①骨髓和（或）组织学确诊的T-ALL/LBL[13]；②符合R/R T-ALL/LBL诊断标准[14]；③年龄≥18岁；④至少有骨髓或一处可测量的病灶用于疗效评价。主要排除标准：①既往化疗含克拉屈滨且治疗无效或虽有效但治疗结束后1个月内疾病进展（PD）；②allo-HSCT后1年内复发。同时纳入自2019年3月至2022年8月在北京大学第三医院血液科行CLAE方案同情用药的患者7例，同情用药患者符合上述主要入选标准①②④，且不符合主要排除标准①。其中4例患者在临床研究开展前行CLAE方案同情用药，3例患者在临床研究开展后不符合入组条件行CLAE方案同情用药（图1）。因所有患者治疗方案及临床管理一致，均纳入分析，通过组间比较更好地探讨CLAE方案的有效性和安全性。本研究经北京大学第三医院伦理委员会批准［批件号：2021医伦理第（059-02）号］，所有患者均签署知情同意书。

**图1:**
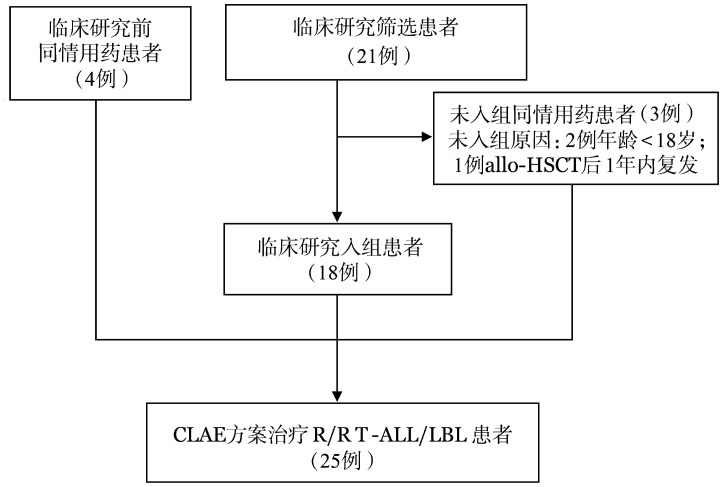
25例难治/复发急性T淋巴细胞白血病/淋巴瘤（R/R T-ALL/LBL）患者来源示意图 **注** allo-HSCT：异基因造血干细胞移植；CLAE：克拉屈滨+阿糖胞苷+依托泊苷

2. 治疗方案：所有患者接受每28天为1个周期的CLAE方案治疗（克拉屈滨5 mg·m^−2^·d^−1^静脉输注，第1～5天；阿糖胞苷200 mg·m^−2^·d^−1^静脉输注，克拉屈滨输注2 h后开始输注，第1～5天；依托泊苷75 mg·m^−2^·d^−1^静脉输注，第1～5天）。计划完成2个周期诱导治疗并进行周期性的疗效和安全性评估。1个周期治疗后PD的患者退出研究，2个周期治疗无效［疾病稳定（SD）或PD］的患者退出研究，治疗有效［CR或部分缓解（PR）］的患者进入巩固治疗，推荐行allo-HSCT，若不满足allo-HSCT条件，经研究者评估可行auto-HSCT或继续CLAE方案巩固。所有患者均随访后续治疗、疗效及生存情况。

3. 疾病相关定义：T-ALL/LBL根据不同的临床表现分为ALL和LBL两种表型，参照2008年WHO第4版造血与淋巴组织肿瘤分类[15]，当骨髓中肿瘤细胞比例≥25％，伴或不伴髓外肿块时，诊断为ALL；当骨髓中肿瘤细胞比例<25％，且以组织肿块为主要临床表现时，诊断为LBL；初诊时具备以下至少1项危险因素定义为高危：年龄>35岁、WBC>100×10^9^/L、早期前体T细胞急性淋巴细胞白血病（ETP-ALL）、RAS/PTEN基因突变和（或）NOTCH1/FBXW7野生型。其余为标危。难治性疾病包括原发难治，即诱导结束时未达到CR；缓解期≤12个月的首次复发；至少二线全身治疗后复发或难治；行allo-HSCT后复发或难治[14],[16]。

4. 疗效评估：参照《中国成人急性淋巴细胞白血病诊断与治疗指南（2016版）》[14]和Lugano淋巴瘤疗效评价标准[17]进行疗效评价：CR：外周血无原始细胞，无髓外白血病；骨髓三系造血恢复，原始细胞比例<5％；外周血ANC>1.0×10^9^/L；外周血PLT>100×10^9^/L；4周内无复发。PR：适用于T-LBL患者，定义为骨髓和外周血达CR，无中枢神经系统白血病（CNSL），肿大的纵隔病灶最大垂直直径的乘积（SPD）缩小50％以上；SD：适用于T-LBL患者，定义为骨髓和外周血达CR，无CNSL，肿大的纵隔病灶改变不满足PR和PD；PD：外周血或骨髓原始细胞绝对数增加25％，或出现髓外疾病；对于T-LBL，任何新发病灶或肿大的纵隔病灶SPD增加≥50％，或PET-CT阳性病灶（Deauville评分为4～5分）伴病灶增大。难治：诱导治疗结束未达CR；复发：已取得CR的患者外周血或骨髓又出现原始细胞（比例>5％），或出现髓外疾病。主要观察终点为2个周期客观缓解率（ORR），为CR率和PR率之和。2个周期后疗效为CR或PR的患者，定义为CLAE缓解组，2个周期后疗效为SD或PD的患者，定义为CLAE未缓解组。次要观察终点为总生存（OS）和无进展生存（PFS）。OS期定义为从接受CLAE方案治疗至因任何原因死亡或末次随访的时间。PFS期定义为从接受CLAE方案治疗到PD或患者死亡或末次随访的时间。

5. 安全性评估：治疗期间监测患者生命体征、不良事件、临床实验室参数（血常规、尿常规、凝血功能、血生化、心电图等）。药物不良反应根据美国国家癌症研究所常见不良反应事件评价标准（CTCAE）5.0版进行报告和分级。

6. 随访：采用查阅门诊/住院病历及电话联系等方式进行随访。所有患者自接受CLAE方案治疗随访至2024年10月31日或死亡，中位随访时间为6.1（1.2～43.7）个月。

7. 统计学处理：人口统计学和临床特征使用描述性统计进行总结分析。计数资料采用例（％）描述，计量资料采用*M*（范围）描述。计数资料组间率的比较采用卡方检验或Fisher精确检验。计量资料采用方差分析及*t*检验或非参数Mann-Whitney *U*检验。生存分析通过Kaplan-Meier方法进行。影响生存的单因素分析采用Log-rank检验。所有统计分析使用Stata 14.0软件和SPSS 22.0软件进行。*P*<0.05为差异有统计学意义。

## 结果

1. 患者临床特征：如表1所示，25例患者中，男性17例（68.0％），确诊T-ALL/LBL的中位年龄29（13～63）岁，临床表型以T-LBL多见（16例，64.0％），ETP亚型11例（44.0％），肿瘤组织Ki-67高表达16例（64.0％），存在纵隔大包块（肿瘤最大直径≥7.5 cm）11例（44.0％）。18例患者可进行预后危险度评估，高危15例（83.3％）。25例患者中，原发难治20例（80.0％），CLAE方案治疗前，中位治疗疗程数6（1～11）个。临床研究组（18例）患者和同情用药组（7例）患者的组间性别、年龄、疾病特征及前期治疗情况差异均无统计学意义（均*P*>0.05）。同情用药组存在纵隔大包块的患者比例较临床研究组高（85.7％对38.9％，*P*＝0.073），且有2例患者为移植后复发。

**表1:** 25例接受CLAE方案治疗的R/R T-ALL/LBL患者的临床特征

临床特征	总体（25例）	临床研究组（18例）	同情用药组（7例）	统计量	*P*值
诊断时年龄［岁，*M*（范围）］	29（13～63）	31（18～63）	26（13～40）	0.761	0.285
性别［例（％）］				0.053	>0.05
男	17（68.0）	12（66.7）	5（71.4）		
女	8（32.0）	6（33.3）	2（28.6）		
ECOG体能评分［例（％）］				0.845	
0～1分	23（92.0）	16（88.9）	7（100）		>0.05
≥2分	2（8.0）	2（11.1）	0（0）		
疾病类型［例（％）］				0.233	>0.05
T-ALL	9（36.0）	7（38.9）	2（28.6）		
T-LBL	16（64.0）	11（61.1）	5（71.4）		
免疫表型［例（％）］				0.682	0.656
ETP	11（44.0）	7（38.9）	4（57.1）		
非ETP	14（56.0）	11（61.1）	3（42.9）		
Ki-67表达［例（％）］				5.297	0.058
≥70％	16（64.0）	14（77.8）	2（28.6）		
<70％	9（36.0）	4（22.2）	5（71.4）		
纵隔大包块（MTD≥7.5 cm）［例（％）］				4.427	0.073
是	11（44.0）	7（38.9）	6（85.7）		
否	14（56.0）	11（61.1）	1（14.3）		
骨髓受累［例（％）］				1.246	0.536
未受累	6（24.0）	5（27.8）	1（14.3）		
白血病细胞比例<25％	10（40.0）	6（33.3）	4（57.1）		
白血病细胞比例≥25％	9（36.0）	7（38.9）	2（28.6）		
中枢神经系统白血病［例（％）］				0.845	>0.05
是	2（8.0）	2（11.1）	0（0）		
否	23（92.0）	16（88.9）	7（100）		
WBC［例（％）］				0.021	>0.05
>100×10^9^/L	4（16.0）	3（16.7）	1（14.3）		
≤100×10^9^/L	21（84.0）	15（83.3）	6（85.7）		
乳酸脱氢酶［例（％）］				2.968	0.177
升高	11（44.0）	6（33.3）	5（71.4）		
正常	14（56.0）	12（66.7）	2（28.6）		
基因突变^a^［例（％）］	9（37.5）	7（41.2）	2（28.6）	0.079	0.961
无突变	3（33.3）	2（28.6）	1（50.0）		
RAS/PTEN突变	3（33.3）	2（28.6）	1（50.0）		
NOTCH1/FBXW7突变	4（44.4）	3（42.9）	1（50.0）		
R/R T-ALL/LBL［例（％）］				0.198	>0.05
原发难治	20（80.0）	14（77.8）	6（85.7）		
复发难治	5（20.0）	4（22.2）	1（14.3）		
既往移植情况［例（％）］				5.590	0.070
未移植	23（92.0）	18（100）	5（71.4）		
auto-HSCT	1（4.0）	0（0）	1（14.3）		
allo-HSCT	1（4.0）	0（0）	1（14.3）		
既往化疗总疗程数［个，*M*（范围）］	6（1～11）	6（1～11）	7（1～10）	0.780	>0.05

**注** CLAE：克拉屈滨+阿糖胞苷+依托泊苷；R/R T-ALL/LBL：难治/复发急性T淋巴细胞白血病/淋巴瘤；ECOG：美国东部肿瘤协作组；ETP：早期前体T细胞；MTD：肿瘤最大直径；WBC：白细胞计数；auto-HSCT：自体造血干细胞移植；allo-HSCT：异基因造血干细胞移植；^a^仅9例患者具有基因突变检测结果，其中1例患者同时携带RAS/PTEN和NOTCH1/FBXW7突变

2. 治疗缓解情况：25例R/R T-ALL/LBL患者CLAE方案治疗前化疗情况、疾病状态、CLAE方案治疗后疗效评估、后续治疗及生存情况见表2。接受Hyper-CVAD（环磷酰胺+长春新碱+多柔比星+地塞米松）方案诱导化疗的患者最多（14例，56.0％），其次为BFM90方案（泼尼松+长春新碱+柔红霉素+左旋门冬酰胺酶）4例、CALLG2008方案（长春新碱+柔红霉素+环磷酰胺+泼尼松+左旋门冬酰胺酶）4例、CHOPE（环磷酰胺+多柔比星+长春新碱+泼尼松+依托泊苷）方案3例。1例入组患者在接受1个周期CLAE方案治疗后顺利出院观察，患者未遵医嘱返院评估及进一步治疗，因电话失联而失访。可评价的24例患者CR率为33.3％（8/24）。

**表2:** 25例接受CLAE方案治疗的R/R T-ALL/LBL患者临床特征、既往治疗情况及治疗后转归情况

例号^a^	性别	治疗时年龄（岁）	诊断	初诊时诱导治疗方案	既往化疗疗程数	接受CLAE方案治疗前疾病状态	1个周期CLAE方案治疗后疗效	2个周期CLAE方案治疗后疗效	后续治疗情况	疾病状态	生存状态^b^
1	男	24	T-LBL	BFM90	6	原发难治/PR	CR	未治疗	allo-HSCT	CR	存活
2	女	18	T-ALL	Hyper-CVAD	8	原发难治/PD	PD	未治疗	出组	PD	死亡
3	女	33	T-LBL	Hyper-CVAD	9	原发难治/PD	PR	CR	allo-HSCT	PD	死亡
4	男	39	T-LBL	CALLG2008	4	复发难治/PD	SD	PD	出组	PD	死亡
5	女	24	T-ALL	Hyper-CVAD	7	原发难治/PR	PD	未治疗	出组，后行CD7 CAR-T细胞治疗后桥接allo-HSCT	CR	存活
6	女	31	T-LBL	Hyper-CVAD	6	原发难治/PD	SD	未治疗	出组	PD	死亡
7	男	27	T-LBL	CHOPE	4	原发难治/PR	PD	未治疗	出组，后行挽救性allo-HSCT	CR	死亡
8	男	63	T-LBL	Hyper-CVAD	7	复发难治/PD	PD	未治疗	出组	PD	死亡
9	男	38	T-LBL	Hyper-CVAD	6	原发难治/PD	PD	未治疗	出组	PD	死亡
10	男	26	T-ALL	Hyper-CVAD	6	原发难治/PD	PD	未治疗	出组	PD	死亡
11	男	18	T-ALL	CHOPE	9	原发难治/SD	CR	CR	allo-HSCT	CR	死亡
12	男	34	T-LBL	CALLG2008	2	原发难治/SD	PR	CR	allo-HSCT	CR	存活
13	男	46	T-ALL	Hyper-CVAD	11	复发难治/PD	CR	未治疗	allo-HSCT	CR	存活
14	男	18	T-LBL	Hyper-CVAD	7	复发难治/PD	CR	CR	allo-HSCT	CR	存活
15	女	18	T-ALL	Hyper-CVAD	6	原发难治/PD	失访	失访	失访	失访	失访
16	男	51	T-LBL	BFM90	2	原发难治/PD	PD	未治疗	出组	PD	死亡
17	女	51	T-LBL	CHOPE	1	原发难治/PD	PD	未治疗	出组	PD	死亡
18	男	18	T-ALL	Hyper-CVAD	4	原发难治/SD	CR	CR	CLAE方案治疗	CR	存活
19	男	27	T-LBL	Hyper-CVAD	10	原发难治/SD	SD	未治疗	放疗	PD	死亡
20	男	42	T-LBL	CALLG2008	7	原发难治/PD	PD	未治疗	化疗	PD	死亡
21	男	35	T-LBL	CALLG2008	4	原发难治/PD	PD	未治疗	CD7 CAR-T细胞治疗	PD	死亡
22	女	26	T-LBL	Hyper-CVAD	10	原发难治/PD	SD	未治疗	CD7 CAR-T细胞治疗	PD	死亡
23	女	34	T-ALL	BFM90	6	原发难治/PD	PD	未治疗	未治疗	PD	死亡
24	男	17	T-LBL	Hyper-CVAD	10	原发难治/PD	PD	未治疗	未治疗	PD	死亡
25	男	13	T-ALL	BFM90	1	复发难治/SD	CR	CR	allo-HSCT	CR	存活

**注** CLAE：克拉屈滨+阿糖胞苷+依托泊苷；R/R T-ALL/LBL：难治/复发急性T淋巴细胞白血病/淋巴瘤；BFM90：柏林-法兰克福-明斯特90，泼尼松+长春新碱+柔红霉素+左旋门冬酰胺酶；Hyper-CVAD：高强度环磷酰胺+长春新碱+多柔比星+地塞米松；CALLG2008：中国成人急性淋巴细胞白血病协作组2008，长春新碱+柔红霉素+环磷酰胺+泼尼松+左旋门冬酰胺酶；CHOPE：环磷酰胺+多柔比星+长春新碱+泼尼松+依托泊苷；PR：部分缓解；PD：疾病进展；SD：疾病稳定；CR：完全缓解；allo-HSCT：异基因造血干细胞移植；CD7 CAR-T细胞：CD7嵌合抗原受体T细胞；^a^例1至例18为临床研究组，例19至例25为同情用药组；^b^生存状态确认时间为2024年10月31日

17例临床研究组患者接受1个周期CLAE方案治疗后ORR为41.2％、CR率为29.4％（5/17）、PR率为11.8％（2/17）；2个周期后评估7例均达CR（其中5例1个周期后达到CR；2例1个周期后达到PR，2个周期后达到CR），骨髓流式细胞术微小残留病（MRD）均为阴性，CR率为41.2％。7例同情用药组患者接受1个周期CLAE方案治疗后仅有1例获得CR，该患者继续巩固1个周期后仍CR，骨髓流式细胞术MRD可见0.1％残留；另外6例患者因SD或PD出组，未行第2个周期CLAE方案治疗，CR率为14.3％。

3. 安全性：本研究采用常见不良事件评价标准（CTCAE）5.0版评估接受CLAE方案治疗的24例患者的不良反应（表3）。结果显示，治疗相关不良反应主要是血液学不良反应。所有患者治疗后均出现了全血细胞减少，其中3级及以上中性粒细胞减少和血小板减少发生率为100％，贫血发生率为83.3％，中位粒细胞缺乏持续时间为7（4～32）d；粒细胞缺乏伴发热发生率为58.3％，其中有2例发生败血症、1例肺感染。非血液学不良反应较轻，主要为轻度的胃肠道反应和转氨酶升高，对症治疗后均改善。治疗期间无治疗相关死亡。

**表3:** 24例接受CLAE方案治疗的R/R T-ALL/LBL患者不良反应发生情况［例（％）］

不良反应类型	总体	3级及以上
中性粒细胞减少	24（100）	24（100）
血小板减少	24（100）	24（100）
贫血	24（100）	20（83.3）
粒细胞缺乏伴发热	14（58.3）	14（58.3）
恶心	11（45.8）	0（0）
呕吐	7（29.2）	0（0）
疲劳	12（50.0）	3（12.5）
AST升高	3（12.5）	0（0）
ALT升高	3（12.5）	0（0）
肌酐升高	1（4.2）	0（0）
败血症	2（8.3）	2（8.3）
肺部感染	1（4.2）	0（0）

**注** CLAE：克拉屈滨+阿糖胞苷+依托泊苷；R/R T-ALL/LBL：难治/复发急性T淋巴细胞白血病/淋巴瘤；AST：天冬氨酸氨基转移酶；ALT：丙氨酸氨基转移酶

4. 后续治疗及生存情况：7例达到CR的临床研究组患者中，排除1例继续行CLAE方案巩固治疗4个周期的CR患者，6例CR患者均接受了allo-HSCT，桥接比例为85.7％（6/7），至随访结束，4例患者持续CR并存活。同情用药组中1例接受CLAE方案治疗后CR，行allo-HSCT后持续CR存活，其余6例CLAE方案治疗无效患者均死亡（表2）。

24例患者中位OS期和PFS期分别为199（46～1 310）d和49（28～1 310）d，6个月、1年、2年累积OS率分别为（52.1±10.2）％、（29.7±9.3）％、（27.1±9.1）％，累积PFS率分别为（32.6±9.6）％、（24.9±8.9）％、（23.8±8.7）％。

17例临床研究组患者中位OS期和PFS期分别为199（46～1 310）d和58（28～1 310）d，6个月、1年、2年累积OS率分别为（50.2±12.1）％、（32.1±11.3）％、（29.4±11.0）％，累积PFS率分别为（35.2±11.6）％、（28.2±12.2）％、（28.2±12.2）％；7例同情用药组患者中位OS期和PFS期分别为161（37～825）d和28（28～825）d，6个月、1年、2年累积OS率分别为（36.4±18.2）％、（14.3±13.2）％、（14.3±13.2）％，累积PFS率分别为（23.4±16.0）％、（14.3±13.2）％、（14.3±13.2）％。

CLAE缓解组共8例患者，7例（87.5％）桥接了allo-HSCT，自治疗至移植的中位时间为75（44～88）d，中位OS期和PFS期均未达到，6个月、1年、2年累积OS率分别为（86.8±12.0）％、（78.3±14.6）％、（72.9±15.7）％，累积PFS率分别为（86.4±12.1）％、（74.8±15.3）％、（72.9±15.7）％；CLAE未缓解组16例患者，中位OS期和PFS期分别为132（37～699）d和28（28～268）d，6个月、1年、2年累积OS率分别为（34.4±11.9）％、（6.3±6.0）％、（6.3±6.0）％，累积PFS率分别为（4.0±4.9）％、0、0。

5. 疗效影响因素：本研究对可能影响CLAE治疗效果的因素进行了单因素分析，结果提示疾病特点、前期治疗情况等均对疗效无影响（均*P*>0.05），年轻男性［诊断时年龄<35岁对≥35岁，41.2％对14.3％，*HR*（95％ *CI*）：0.238（0.023～2.440），*P*＝0.352；男性对女性，41.2％对14.3％，*HR*（95％ *CI*）：0.238（0.023～2.440），*P*＝0.352］、T-ALL表型［T-ALL对T-LBL，50.0％对25.0％，*HR*（95％ *CI*）：0.333（0.056～1.995），*P*＝0.363］、无纵隔大包块［无纵隔大包块对纵隔大包块，45.5％对23.1％，*HR*（95％ *CI*）：0.360（0.062～2.078），*P*＝0.390］、复发难治［复发难治对原发难治，60.0％对26.3％，*HR*（95％ *CI*）：0.238（0.030～1.868），*P*＝0.289］的患者ORR相对较高，但差异均无统计学意义。

## 讨论

本研究R/R T-ALL/LBL患者接受CLAE方案治疗的再诱导CR率为33.3％，其中临床研究组CR率达41.2％，与现有文献报道的奈拉滨、维奈克拉及Hyper-CVAD、MOpAD（甲氨蝶呤+长春新碱+培门冬酶+地塞米松）等方案疗效相当[1]–[2],[18]–[24]。本研究87.5％的CR患者成功桥接allo-HSCT，移植后最长无病生存期已达42个月，提示该方案可为后续根治性治疗创造关键窗口期。

目前R/R T-ALL/LBL的治疗无统一方案，面临再诱导疗效有限与治疗相关不良反应发生风险两大挑战[1]–[2]。T-ALL/LBL分子分型的进展和新型免疫治疗有望改善治疗现状[4],[7],[25]。髓系肿瘤和急性白血病的国际共识分类（ICC）在ETP-ALL诊断中新增了“BCL11B激活型”的亚型，并根据异常激活的转录因子家族在急性T淋巴细胞白血病，非特指型分类中新增8个临时诊断[26]，为潜在的分子靶向治疗提供依据[4],[7]。而以CD38、CD52为代表的免疫治疗[27]和以CD7、CD5为代表的CAR-T细胞治疗[27]–[28]在R/R T-ALL/LBL中显现出良好的前景，但目前多处于早期临床研究阶段，疗效和安全性有待进一步明确。

R/R T-ALL/LBL的再诱导治疗可选择的方案有限且缓解率低、中位OS期短[1]–[2]。含奈拉滨方案被指南推荐用于治疗R/R T-ALL/LBL[1]–[2]。DeAngelo等[18]和Candoni等[19]报道奈拉滨单药治疗成人R/R T-ALL/LBL患者的CR率分别为31％和36％，1年OS率仅为28％和22％，8％的患者发生3级以上神经毒性[19]。Luskin等[20]报道NCE（奈拉滨+环磷酰胺+依托泊苷）方案治疗5例首次复发的成人T-ALL/LBL，其中3例获得CR，2例死于化疗相关并发症。含奈拉滨方案疗效欠佳，安全性低，且奈拉滨未在国内上市。克拉屈滨作为脱氧腺苷类似物，具有多种抗肿瘤机制，与阿糖胞苷具有协同增效作用，在白血病和淋巴瘤的治疗中具有良好的疗效和安全性[8]–[11]。研究表明克拉屈滨联合化疗治疗复发难治进展期T细胞和NK细胞淋巴瘤后桥接allo-HSCT可提高疗效[12]。本研究采用CLAE方案治疗R/R T-ALL/LBL，临床可及性和安全性良好，再诱导治疗缓解后的患者桥接allo-HSCT，疗效确切。

指南推荐的治疗R/R T-ALL/LBL其他化疗方案包括Hyper-CVAD方案、MOpAD方案、维奈克拉联合化疗等[1]–[2]。德克萨斯大学门罗·杜纳维·安德森癌症中心采用强化的Hyper-CVAD方案治疗90例成人R/R ALL，其中18例为R/R T-ALL/LBL[21]。88例可评价患者的总缓解率为47％，19例（21％）桥接allo-HSCT，治疗期间共15例（17％）患者死亡，其中8例在治疗30 d内死亡，12例死于重症感染，中位PFS期和OS期分别为6个月和6.5个月[21]。Kadia等[22]报道MOpAD方案治疗37例R/R ALL，其中11例R/R T-ALL有5例CR，是否桥接移植未作说明，2例在6个月内复发。研究期间共14例患者死亡，9例死于各种感染，30 d内死亡率高达19％[22]。上述方案虽然显示出一定的疗效，但治疗相关死亡率尤其是早期死亡率高。Richard-Carpentier等[23]报道维奈克拉联合化疗治疗13例成人R/R T-ALL，CR率为40％，但仅1例患者桥接allo-HSCT；8例有初始疗效的患者6例复发；所有13例患者中位随访10.4个月，6例患者死亡，中位OS期和无复发生存期分别为7.7个月和4个月，1年OS率为44％。以上提示再诱导缓解后未桥接移植，早期复发率高。国内一项回顾性研究报道了维奈克拉联合化疗治疗15例R/R ETP-ALL的疗效，10例CR，其中8例桥接了allo-HSCT，7例存活[24]。所有患者中位OS期为17.7个月，1年OS率为60％，桥接移植的患者生存优于未桥接移植患者［中位OS期：未达到对2（0～13）个月，*P*＝0.009］[24]。本研究采用CLAE方案再诱导治疗R/R T-ALL/LBL，CR率为33.3％，临床研究组CR率为41.2％，疗效与文献报道相近[21]–[24]。CLAE方案治疗期间患者虽均出现了Ⅳ度骨髓抑制，但中位粒细胞缺乏时间仅为7 d，造血恢复快，无感染及其他治疗相关死亡，安全性优于文献报道[21]–[22]。本研究中CR患者桥接allo-HSCT的比例达87.5％，高于文献报道[21],[23]–[24]，为患者长期生存获益奠定了基础。

然而，各研究的R/R T-ALL/LBL例数较少，且不同研究入组患者临床特征及随访时间存在差异，难以进行不同再诱导方案之间疗效和安全性的直接比较。本研究纳入的R/R T-ALL/LBL患者在以原发难治（80％）、初诊高危（83.3％）为主的基线条件下，获得了与既往研究相当的CR率，表明CLAE方案可作为无法入组新药临床试验患者的有效替代选择。多个研究均表明R/R T-ALL/LBL再诱导缓解后缓解期短，需尽快桥接allo-HSCT实现长期缓解生存[21]–[24]。CLAE方案治疗R/R T-ALL/LBL具有快速诱导缓解和不良反应发生率低的双重优势，使患者有效桥接allo-HSCT，促进实现长期生存。

综上所述，CLAE方案作为一种具有较高临床可及性和可行性的化疗方案，治疗R/R T-ALL/LBL安全有效，可使患者获得CR后行allo-HSCT治疗，改善患者预后。本研究样本量较小，未发现影响疗效的因素，未能进行多因素分析，随访时间较短，长期生存数据尚需延长随访时间验证。目前结果显示，临床研究组患者CR率和生存优于同情用药组患者，而同情用药组纵隔大包块、原发难治患者多，可能是其疗效不佳的原因；CLAE方案治疗后CR可改善长期生存。后续需进一步扩大样本量，进一步证实CLAE方案的疗效和安全性并寻找其影响因素，选择出更能从CLAE方案获益的患者，提高患者的再诱导缓解率并积极桥接allo-HSCT，改善患者的长期生存。
